# Supplementary material for: Functional profiling of 2,193 ASS1 missense variants: Insights into variant pathogenicity and epistatic interactions in citrullinemia type I
Source: PLoS Genet. 2026 Jun 17;22(6):e1012167. doi: 10.1371/journal.pgen.1012167 (PMC13289927; doi:10.1371/journal.pgen.1012167)
Supplement: S1 Fig — Single-nucleotide variant heatmap where each ASS residue is represented by 5-7 variants. (DOCX) [file pgen.1012167.s001.docx]

**S1 Fig**


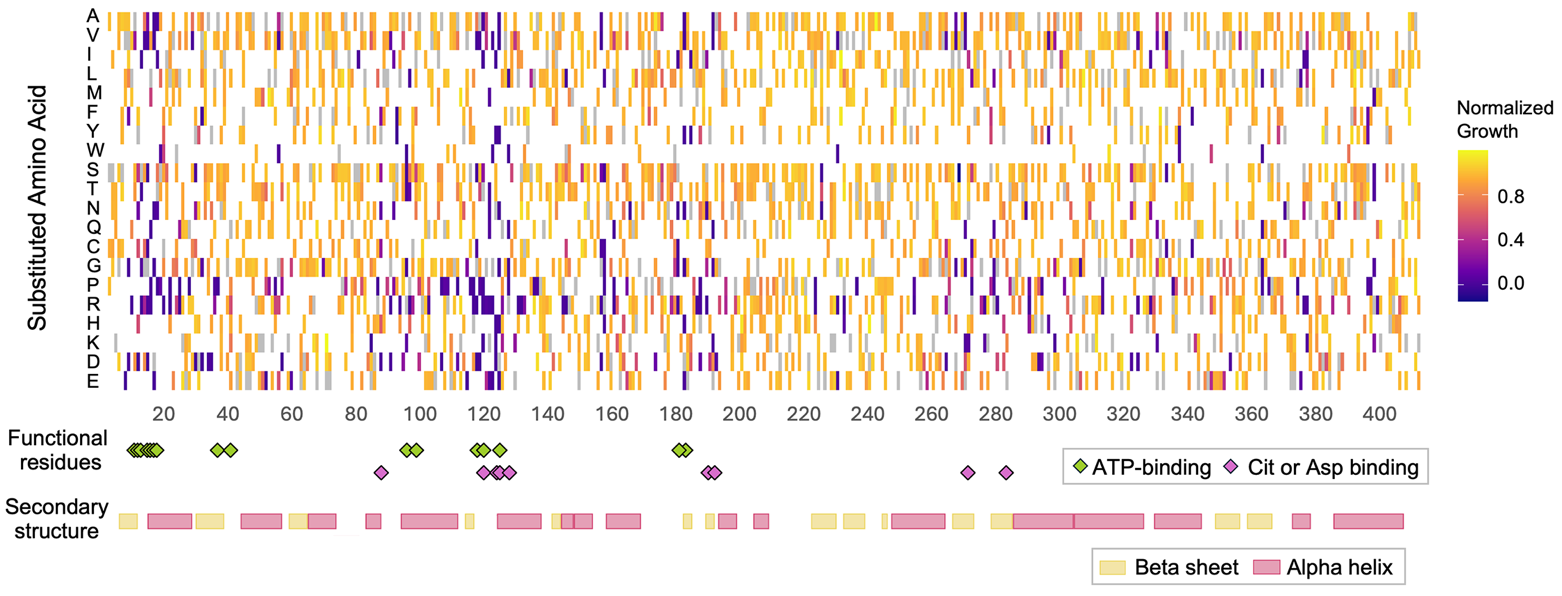


**S1 Fig. Heatmap of SNV Variant Normalized Yeast Growth**

Single-nucleotide variant heatmap where each ASS residue is represented by 5-7 variants.
