# Supplementary material for: Functional profiling of 2,193 ASS1 missense variants: Insights into variant pathogenicity and epistatic interactions in citrullinemia type I
Source: PLoS Genet. 2026 Jun 17;22(6):e1012167. doi: 10.1371/journal.pgen.1012167 (PMC13289927; doi:10.1371/journal.pgen.1012167)
Supplement: S2 Fig — (DOCX) [file pgen.1012167.s002.docx]

**S2 Fig**


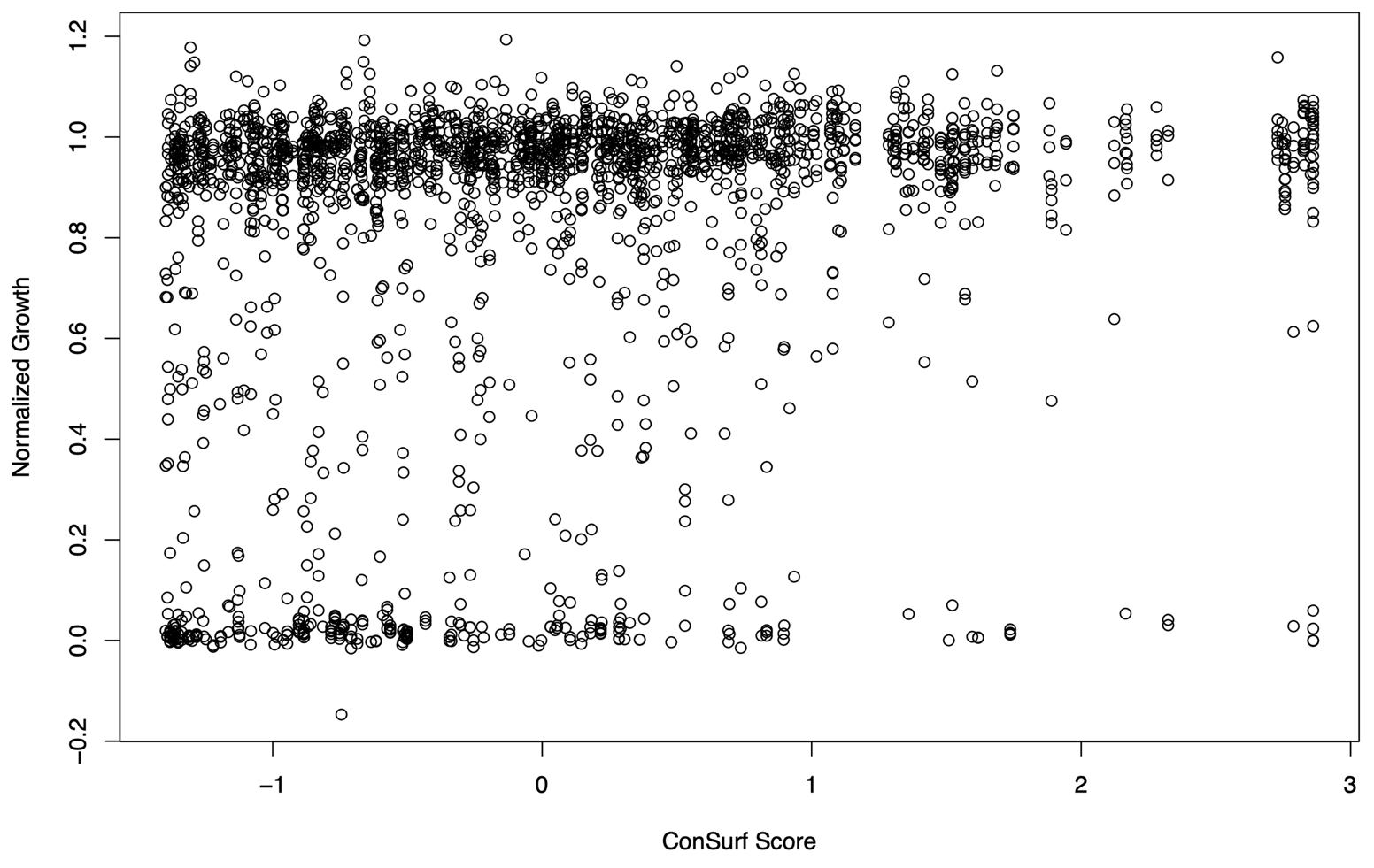


**S2 Fig. Normalized growth score of each variant is plotted against the conservation (ConSurf score) of the corresponding residue**
